# Supplementary material for: WordCommentsAnalyzer: A windows software tool for qualitative research
Source: F1000Res. 2018 Sep 4;7:536. Originally published 2018 May 3. [Version 2] doi: 10.12688/f1000research.14819.2 (PMC6137411; doi:10.12688/f1000research.14819.2)
Supplement: Supplementary file 3 [file f1000research-7-17614-s0002.tgz › b2a79496-4ada-47c4-9682-a99ce867836d.zip!/Eight Iranian Health Journals/Yafteh _23.docx]

© Yafteh  . This work is licensed under CC BY 4.0 (https://creativecommons.org/licenses/by/4.0/).

Volume & Issue: دوره 14 - شماره 5

Title: بررسی مواجهه شغلی با خون و مایعات بدن بیماران و عوامل مرتبط با آن در پرسنل شاغل در بیمارستان شهدای عشایر شهر خرم آباد

http://yafte.lums.ac.ir/article-1-1106-fa.html

مقدمه: پرسنل رشته‌های مختلف پزشکی به اقتضای حرفه خود، همواره در معرض آلودگی با خون و مایعات بدن بیماران هستند. هدف این مطالعه بررسی مواجهه شغلی با خون و ترشحات بیماران در پرسنل بیمارستان عشایر شهر خرم آباد بوده است. مواد و روش‌ها: در این مطالعه مقطعی، در طی سالهای 1389و1390 کلیه پرسنل شاغل در بیمارستان شهدای عشایر که با خون و سایر ترشحات بدن بیماران مواجهه یافته بودند، مورد بررسی قرار گرفتند. ابزار گردآوری اطلاعات پرسشنامه‌ای شامل ویژگی‌های جمعیت شناختی افراد مواجهه ‌یافته، علل مواجهه، راه مواجهه، منبع مواجهه و وضعیت واکسیناسیون هپاتیت B بود. اطلاعات با استفاده از آمار توصیفی و آزمون دقیق فیشر مورد تجزیه و تحلیل قرار گرفت. بحث و نتیجه‌گیری: آموزش شیوه‌های پیشگیری از مواجهه با ترشحات بیماران همراه با استفاده از وسایل حفاظتی و تقویت سطح مهارت‌های بالینی پرسنل بیمارستانی، می‌تواند در پیشگیری از مواجهه‌های شغلی در بیمارستانها نقش بسزایی داشته باشد. یافته‌ها: در فاصله زمانی ذکر شده، 80 نفر از پرسنل بیمارستان با خون و ترشحات بیماران مواجهه یافته بودند. بیشترین مواجهه یافتگان پرستاران (5/32%) و سپس دانشجویان (20%) و کارورزان پزشکی (3/11%) بودند. بیشترین مواجهه در بخش اورژانس (3/41%) رخ داده بود. در 3/96% موارد مواجهه از طریق پوست بود و اکثر موارد مواجهه (5/52%) در شیفت صبح گزارش شده بود. بیشترین علت مواجهه رگ گیری (3/26%) و غلاف کردن سوزن (5/22%) بود. . منبع آلودگی در 3/56% موارد نامشخص بود. بین علل مواجهه با ترشحات بیماران بر اساس جنس و نوع حرفه فرد تفاوت آماری معناداری دیده شد (05/0(P<.

Title: مقایسه سطوح چربی‌های سرم در مادران باردار مبتلا به دیابت بارداری با خانم‌های باردار طبیعی

http://yafte.lums.ac.ir/article-1-1107-fa.html

مقدمه: اگرچه تصور می‌شود که تغییرات هورمونی در بارداری سبب تغییر در سطوح سرمی چربی‌ها می‌شود اما هنوز مکانیسمی که حاملگی موجب تغییر متابولیسم چربی‌ها می‌شود ناشناخته است. بحث و نتیجه‌گیری: بر اساس ارتباط مثبت بین هیپرتری‌گلیسیریدمی و هیپرانسولینمی این احتمال وجود دارد که افزایش سطح تری گلیسیرید در موارد دیابت بارداری انعکاسی از تغییرات سطح انسولین در سرم مادر باشد. یافته‌ها: سطوح گلوکزسرم،توتال کلسترول، کلسترول با دانسیته پایین (LDL-C ( و کلسترول با دانسیته بالا (HDL-C) در بین دو گروه تفاوت معنی‌داری نشان نداد. درحالی‌که مقادیر انسولین، اندکس مقاومت به انسولین (HOMA-IR) و سطوح تری‌گلیسیرید در خانم‌های مبتلا به دیابت بارداری بخصوص بعد از هفته 32 حاملگی به صوررت معنی‌داری بالاتر بود (05/0< P) و بر اساس آزمون آماری اسپیرمن افزایش سطح تری‌گلیسیرید با افزایش مقاومت به انسولین و افزایش HOMA-IR همراه بود (001/0=P). مواد و روش‌ها: این مطالعه به صورت آینده نگر، بر روی 112 خانم باردار مبتلا به دیابت بارداری و 159 خانم باردار سالم دربیمارستان شریعتی درفاصله زمانی اسفند1389 تا اردیبهشت 1391 انجام شد. به منظورتعیین تغییرات لیپید ولیپوپروتئین‌هادر بارداری سطوح اجزای لیپیدها، گلوکز و انسولین در سرم مادر در سه نوبت در سه ماهه سوم اندازه‌گیری شد.

Title: بررسی اثر ضد درد شیر مادر در مقایسه با شیر مصنوعی و دکستروز50 % هنگام خونگیری وریدی

http://yafte.lums.ac.ir/article-1-1108-fa.html

بحث و نتیجه‌گیری: شیر دوشیده شده به صورت ﻣﺆثری درد در هنگام خونگیری وریدی را در نوزادان کاهش می دهد و می تواند به عنوان یک ضد درد طبیعی، غیر تهاجمی و در دسترس به ویژه هنگامی که مادر قادر به حضور در بخش نوزادان جهت شیردهی نیست، مورد استفاده قرار گیرد . مواد و روش‌ها: این مطالعه یک کار آزمایی بالینی بود که در آن 30 نوزاد ترم مورد بررسی قرار گرفتند. هر نوزاد قبل از هر یک از سه نوبت خونگیری متوالی، شیر دوشیده شده مادر، شیر خشک و دکستروز 50% را به صورت تصادفی و بدون ترتیب خاصی دریافت کرد و پاسخ رفتاری وی به درد با استفاده از معیارهای امتیاز بندی درد در نوزادانDAN Score) )اندازه گیری و ثبت گردید. مطالعه به صورت دو سویه کور انجام شد و مشاهده گر از نوع ماده تجویز شده کاملاً بی اطلاع بود . یافته‌ها: نتایج حاصل از این پژوهش نشان داد نمره درد به طور معنی داری در نوزادانی که شیر دوشیده شده مادر دریافت کردند نسبت به دریافت کنندگان شیر خشک و دکستروز 50% کمتر بود . دریافت کنندگان شیر خشک بیشترین نمره درد را داشتند . جنس ، سن و وزن نوزاد تاثیری در پاسخ به درد نداشتند . مقدمه: نوزادان حتی وقتی سالم متولد می شوند ممکن است تحت اقدامات تهاجمی و درناک قرار بگیرند. شواهدی وجود دارد که نوزادان به خوبی درد را احساس می کنند و حساسیت و عوارض طولانی مدت به درد در آنها بیش از کودکان بزرگ تر است. درمان های غیر دارویی برای کاهش درد هنگام اقدامات دردناک نوزادان ارزشمند می باشد. هدف از این پژوهش بررسی اثر ضد درد شیر دوشیده شده مادر در کاهش درد نوزادان حین خونگیری وریدی و مقایسه آن با شیر خشک و دکستروز 50% می باشد.

Title: شناسایی الگوهای غذایی غالب در بزرگسالان ایرانی

http://yafte.lums.ac.ir/article-1-1109-fa.html

مقدمه: شناسایی الگوهای غذایی دارای اهمیت زیادی است. تا آنجا که ما می‌دانیم تا کنون مطالعه ای در این زمینه در غرب کشور صورت نگرفته است. هدف از این مطالعه تعیین الگوهای غذایی غالب در بزرگسالان شهر خرم آباد بود. مواد و روش‌ها: در این مطالعه مقطعی رژیم غذایی 973 نفر( 736 زن) از بزرگسالان شهر خرم آباد با استفاده از پرسشنامه تکرر مصرف مواد غذایی208 موردی بررسی شد. این اقلام غذایی به 40 گروه غذایی طبقه بندی شدند و با تحلیل عاملی در برنامه SPSS مورد تجزیه و تحلیل آماری قرار گرفتند. بحث و نتیجه‌گیری: سه الگوی غذایی در بزرگسالان 18 تا 70 ساله شهر خرم آباد شناسایی شد. ارتباط این الگوهای غذایی با مشکلات مربوط به سلامت در مطالعه دیگری بحث خواهد شد. یافته‌ها: تحلیل عاملی سه الگوی غذایی غالب را مشخص نمود: الگوی غذایی سالم( ماکیان، لبنیات کم چرب و پر چرب، سبزیجات کلمی، زرد و سبز تیره، سایر سبزیجات، حبوبات، غلات کامل، ماهی، زیتون و میوه)، الگوی غذایی غربی ( گوشت قرمز و فراوری شده، گوشت احشاء، مارگارین، چاشنی‌ها، دسرها، نوشابه، میان وعده، خشکبار، شیرینی و دسر) و الگوی غذایی سنتی(تخم مرغ، چای، آب میوه، غلات تصفیه شده، مغزها، ترشی، روغن جامد، قند و شکر، نمک و ادویه جات). در کل این الگوهای غذایی 88/29% از واریانس را توجیه می کردند. تفاوتی بین مردان و زنان از نظر نوع الگوی غذایی مشاهده نشد.

Title: عوامل مرتبط باکاهش رشد قد و وزن در شیر خواران زیر 2 سال شهرستان الشترلرستان

http://yafte.lums.ac.ir/article-1-1110-fa.html

مقدمه: رشد کودکان یکی از مهم‌ترین منابع اطلاعاتی برای تشخیص در تأخیر روند رشد و سوء تغذیه در کودکان است. هدف از این مطالعه بررسی عوامل مرتبط با کاهش رشد قد و وزن در شیرخواران زیر 2 سال شهرستان الشتر لرستان بود. مواد و روش‌ها: این بررسی یک مطالعه مقطعی بود. جامعه آماری مورد مطالعه کلیه متولدین زیر 2 سال مراجعه کننده به مراکز بهداشتی شهرستان الشتر در نیمه اول سال 1386 بود. کلیه اطلاعات توسط پرسشنامه جمع‌آوری و توسط نرم‌افزار آماری SPSS مورد تجزیه قرار گرفت. بحث و نتیجه‌گیری: در این مطالعه 8/25 درصد از شیرخواران کاهش در منحنی رشد وزن و 8 درصد کاهش در منحنی رشد قد داشتند. بین کاهش منحنی قد و وزن با متغیرهایی مانند محل سکونت شیرخوار، بیماری‌های عفونی، سطح تحصیلات مادر، وضعیت اشتغال مادر، شغل پدر، استفاده از مکمل آهن و وزن کم موقع تولد ارتباط معنی‌دار وجود دارد. یافته‌ها: از 299 شیرخوار مورد بررسی 77 نفر (8/25 درصد) کاهش در منحنی رشد وزن و 24 نفر (8 درصد) کاهش در منحنی رشد قد داشتند. بین کاهش منحنی رشد وزن با متغیرهایی مانند محل سکونت شیرخوار، بیماری‌های عفونی، شیر مصرفی، مدت زمان تغذیه با شیر مادر، زمان آغاز تغذیه تکمیلی، فاصله تولد بعدی، سطح تحصیلات مادر، وضعیت اشتغال مادر، شغل پدر، استفاده از مکمل آهن و وزن کم موقع تولد ارتباط معنی‌دار وجود دارد. بین کاهش منحنی رشد قد با محل سکونت، بیماری‌های عفونی، مدت زمان تغذیه با شیر مادر، استفاده از مکمل آهن و ویتامین آ+د، وزن کم موقع تولد، سطح تحصیلات مادر، وضعیت اشتغال مادر و شغل پدر ارتباط معنی‌دار وجود دارد.

Title: بررسی کارایی فرآیند فتوکاتالیستی نانوذرات ZnO بر روی رنگبری متیلن بلو و حذف COD از فاضلاب سنتتیک

http://yafte.lums.ac.ir/article-1-1111-fa.html

مقدمه: پسابهای حاوی رنگهای سنتتیک برای سیستم‌های اکولوژیکی و سلامت عمومی خطرناک هستند. متیلن بلو یک رنگ شیمیایی آروماتیک پرکاربرد و مهم در صنایع نساجی است. با توجه به آروماتیک بودن اغلب سمی، سرطان زا و جهش زا است. هدف از انجام این مطالعه بررسی کارایی فتوکاتالیستی نانوذرات اکسید روی جهت رنگبری متیلن بلو توسط پرتوهای UV از فاضلاب سنتتیک نساجی می‌باشد. مواد و روش‌ها: در این پژوهش تجزیه فتوکاتالیستی رنگ متیلن بلو با استفاده از نانو ذرات اکسید روی با قطر کمتر از 50 نانو متر و پرتو فرابنفش (UVC) در یک راکتور ناپیوسته از جنس پلکسی گلاس مورد مطالعه قرار گرفت. راکتور از دو قسمت تشکیل شده بود که یک قسمت آن شامل نانو ذرات تثبیت شده بر روی شیشه و محلول حاوی رنگ و قسمت دوم محل قرارگیری لامپ‌های UV بود. زمان تماس، غلظت رنگ، شدت تابش و فاصله لامپ تا سطح کاتالیست از جمله متغیرهای مورد بررسی در این تحقیق بودند. بحث و نتیجه‌گیری: با توجه به آزمایش های انجام شده، نتایج این تحقیق نشان داد که این روش (فرآیند فتوکاتالیتیکی اکسید روی) با بهینه سازی عوامل عملیاتی موثر، کارایی مناسبی در رنگبری ماده رنگزای متیلن بلو و حذف COD دارد و می توان در مقیاس های بزرگ تر انجام داد. یافته‌ها: خصوصیات مربوط به نانو ذرات اکسید روی بوسیله میکروسکوپ الکترونی روبشی (SEM) و آنالیز XRD مشخص گردید. تصاویر نشان می دهند که با تثبیت کردن نانو ذرات بر روی شیشه، میزان تخلخل آنها در حد مطلوبی باقی مانده است. زمان تماس 120 دقیقه، غلظت بهینه رنگ mg/l 25، شدت تابش بهینه μW/cm2 3950 و فاصله بهینه لامپ تا سطح کاتالیست cm1 حاصل شد. راندمان رنگبری متیلن بلو و حذف COD در مقادیر بهینه کلیه متغیرها به ترتیب 2/92 و 75 درصد بدست آمد.

Title: بررسی اثر دگزامتازون بر بیان گالکتین-3 در سلول‌های زایای اسپرم موش سوری

http://yafte.lums.ac.ir/article-1-1112-fa.html

مقدمه: مطالعات قبلی نشان داده‌اند که دگزامتازون یک داروی گلوکوکورتیکوئیدی پر مصرف است که با کاهش سطوح تستوسترون باعث ایجاد آپوپتوز در سلول‌های زایای اسپرم می‌شود. گالکتین-3 (Gal-3)، نیز که عضوی از خانواده لکتین‌های بتاگالاکتوزیدی است، نقش‌های مهمی در وقایع بیولوژیک مختلف مانند رشد سلولی، آپوپتوز، چسبندگی و تعدیل ایمنی ایفاء می‌کند. در این مطالعه اثر دگزامتازون بر بیان Gal-3 در بافت بیضه موش سوری بررسی شده است. بحث و نتیجه‌گیری: این یافته‌ها نشان می‌دهند که دگزامتازون احتمالاً با کاهش بیان Gal-3 باعث ایجاد آپوپتوز در سلول‌های زایای اسپرم موش می‌شود. یافته‌ها: در گروه کنترل Gal-3در تمام مراحل چرخه اسپرماتوژنز به‌ویژه مراحل VII-VIII بیان شده بود. در گروه تیمار شده با دگزامتازون بیان Gal-3 در سلول‌های زایا کاهش یافته بود. الگوی بیان Bcl-2 در گروه‌های کنترل و آزمون مشابه گالکتین-3 بود. در بررسی HSCORE بیان Gal-3 و Bcl-2 در سلول‌های زایا در تمام مراحل چرخه اسپرماتوژنز کاهش معنی داری را نشان داند (01/0 P=). در بررسی‌های تانل ایندکس آپوپتوز سلول‌های زایا در گروه آزمون افزایش معنی داری نشان داد. مواد و روش‌ها: 16 موش سوری نر بالغ (6 -8 هفته) با نژاد NMRI به‌طور تصادفی به دو گروه تقسیم شدند. گروه آزمون mg/kg 7 دگزامتازون به‌مدت 7 روز و به‌صورت تزرق داخل صفاقی در یافت کرد. به گروه کنترل سرم فیزیولوژی به‌مدت 7 روز و به‌صورت تزریق داخل صفاقی داده شد. یک روز پس از آخرین تزریق حیوانات قربانی شده و سپس بیضه‌های آنها در فرمالین 10% فیکس شد. برای بررسی بیان Gal-3 و Bcl-2 و آپوپتوز از آزمایشات ایمنوهیستوشیمی و تانل استفاده شد. برای درجه بندی و مقایسه نتایج ایمنوهیستوشیمی از روش HSCORE استفاده شد.

Title: جداسازی و شناسایی باکتری های تجزیه کننده فنل از رودخانه کر و بررسی سینتیک رشد آنها

http://yafte.lums.ac.ir/article-1-1113-fa.html

مقدمه: درگذشته از روش های فیزیکوشیمیایی برای حذف فنل ومشتقات آن استفاده می شد ولی امروزه تصفیه زیستی در اولویت قرار دارد. باکتری ها با تکثیر سریع در حضور فنل و ترکیبات آن توانایی فوق العاده ای را در حذف این ترکیبات نشان داده اند .هدف از این پژوهش جداسازی و شناسایی باکتری های تجزیه کننده فنل ازآب و رسوب رودخانه کر و بررسی سینتیک رشد آنها در حضور این ماده سمی می باشد. مواد و روش‌ها: 60 نمونه آب و رسوب از مناطق مختلف رودخانه کر جمع آوری گردید. جداسازی باکتری های تجزیه کننده فنل با کشت نمونه ها بر روی محیط پایۀ نمکی فنل براث انجام شد. برای غربالگری باکتری های تجزیه کننده فنل معرف برموتیمول بلو به محیط اضافه گردید. در نهایت با کشت باکتری ها در غلظت های مختلف فنل، که از 2/0 تا 9/. گرم برلیتر بود، توانایی باکتری ها در تجزیه غلظت های مختلف فنل اندازه گیری شد. به منظور بررسی سینتیک رشد باکتریها از ثبت مداوم 600OD استفاده گردید. در این روش پس از ثبت اولیه جذب نوری، محیطهای کشت در دمای 0C30 به مدت 24 ساعت در انکوباتور شیکردار و با دور rpm 100 قرار داده شد و تا 300 ساعت جذب نوری قرائت گردید. بحث و نتیجه‌گیری: رودخانه کر دارای تعداد زیادی از باکتری های تجزیه کننده فنل است که قدرت تجزیه ای بالایی دارند. از مهمترین این جنس ها می توان به سودوموناس و اسینتوباکتر اشاره کرد. یافته‌ها: سودوموناس ها از جمله مهمترین باکتری های تجزیه کننده فنل جداشده از رودخانه کر بودند که فراوانی وسیعی را در قسمت های مختلف رودخانه نشان دادند. اسینتوباکتر نیز به نوبۀ خود قابل توجه بود. اکثر باکتری های جدا شده قدرت قابل ملاحظه ای در تجزیه فنل نشان دادند. به طوری که سودوموناس تا غلظت 9/0 گرم برلیتر, اسینتوباکتر تا غلظت 8/0 گرم بر لیتر، شیگلا، سالمونلا، کلبسیلا و سیتروباکتر تا غلظت حدود 6/0 گرم بر لیتر و بقیه باکتری ها تا غلظت 3/0-2/0 گرم بر لیتر فنل را توانستند حذف کنند. بررسی میزان 600OD نیز تائیدی بر نتایج به دست آمده بود به طوری که بالاترین میزان 600OD مربوط به باکتری سودوموناس به دست آمد و ماکزیمم جذب نوری هر باکتری در حداکثر غلظت تجزیه به وسیله آن مشاهده گردید.

Title: بهینه سازی سیستم اطلاعات الکترونیکی بیمارستانی دربخش ویژه قلبی: تحقیق عملکردی مشارکتی

http://yafte.lums.ac.ir/article-1-1114-fa.html

مقدمه: یکی ازشاخص‌های توسعه بهداشتی درمانی وجود سیستم‌های اطلاعات الکترونیکی بیمارستانی(HIS) است، اما از فرایند پرستاری به عنوان یک روش علمی پشتیبانی نمی‌کند. بنابراین پژوهش حاضر با هدف بهینه سازی HIS دربخش ویژه قلبی بارویکرد"مراقبت پرستاری باروش علمی" انجام شد. بحث و نتیجه‌گیری: با دسترسی آسان به استانداردهای مراقبت پرستاری، استفاده بهینه از HIS مهیا شد. پیشنهاد می‌شود با ارتقاء توانمندی‌های HIS و ارتباط چند رشته‌ای، دریافت مراقبت‌های علمی و ایمن، و ثبت دقیق و سریع با استفاده از تکنولوژی فراهم شود. یافته‌ها: با بهینه سازی HIS، میانگین مراقبت پرستاری بر بالین بیمار در حیطه های مختلف و در کل از 40/177 به06/ 273 افزایش یافت (001/0P= ). همچنین رابطه پرستار با بیمار و تیم مراقبتی بهتر شده و دستاورد آن، راحتی خیال بیمار، اطمینان به مراقبت‌های دریافتی، اعتماد به پرستار، کاهش شکایات و افزایش رضایتمندی بیمار و همراهان بود. مواد و روش‌ها: این مطالعه بخشی از یک تحقیق عملکردی مشارکتی است که در بخش مراقبت ویژه قلبی مرکز آموزشی درمانی شهید مدنی خرم آباد در سال‌های 1389-1390 در چهار فاز بررسی وضع موجود، طراحی، اجرا واررزشیابی برنامه‌های تغییر انجام شد. کلیه کسانی که در بکار گیری HIS تاثیر گذار بودند به عنوان شرکت کننده انتخاب شدند(44 نفر). روش جمع آوری داده ها مصاحبه باز، بحث متمرکز گروهی، جلسات گروه هدایت کننده، چک لیست مشاهده مراقبت پرستاری بر بالین بیماربود. نیاز به تغییر در نحوه ثبت گزارشات پرستاری درHIS تبیین، تایید وطراحی شد. مراقبت های پرستاری به روش SOAPIE، تهیه و با همکاری واحدIT در HISقرار داده شد و برنامه ها اجرا و ارزشیابی گردید. همچنین انعکاس و به شور گذاشتن برای اعتبار بخشی استفاده ‌شد. آنالیز داده ها به روش مقایسه مداوم و آزمون ویلکاکسون انجام گردید.

Title: تاثیر مکمل یاری ترکیبی آنتی اکسیدانی بر میزان چربی‌های سرمی در زنان مبتلا به آرتریت روماتوئید

http://yafte.lums.ac.ir/article-1-1115-fa.html

بحث و نتیجه‌گیری: تجویز روزانه مکمل ترکیبی آنتی اکسیدانی در زنان مبتلا به RA به مدت سه ماه می تواند باعث بهبود وضع چربی‌های سرم شده و احتمالا خطر ابتلا به آترواسکلروزیس را در آینده کاهش دهد. مواد و روش‌ها: در یک مطالعه قبل-بعد، 40 زن مبتلا به آرتریت روماتوئید یک کپسول سلن‌پلاس (سلنیم 50 میکروگرم، روی 8 میلیگرم، ویتامین A400 میکروگرم، ویتامینC 125 میلیگرم و ویتامینE 40 میلیگرم) را روزانه به مدت سه ماه دریافت کردند. قبل و بعد ازمداخله، 5 میلی‌لیتر خون وریدی ناشتا از بیماران اخذ و سطح سرمی کلسترول‌تام(TChol)، تری‌گلیسرید(TG) و لیپوپروتئین ‌با دانسیته ‌بالا(HDL-c) با کیت‌های آنزیمی و سطح لیپوپروتئین ‌با دانسیته‌پایین(LDL-c) با فرمول فریدوالد محاسبه گردید. به منظور آنالیز آماری از نرم افزارSPSS نسخه5/13برای تست توزیع نرمال از Shapiro-wilk test و همچنین از هیستوگرام با منحنی توزیع نرمال و برای اختلاف قبل-بعد ازتست t زوجی استفاده شد. یافته‌ها: از 40 بیمار، 39 نفر در مطالعه باقی ماندند. مقادیر سرمی کلسترول تام، تری‌گلیسرید، نسبتTG/HDL-c و LDL-c در مقایسه به قبل کاهش معنی‌داری یافتند (بترتیب001/0< P، 001/0< P، 001/0< P،01/0< P)، در حالی‌که HDL-c و HDL-c/LDL-c نسبت اختلاف معنی‌داری نداشت. مقدمه: آرتریت روماتوئید(Rheumatoid arthritis) از شایعترین بیماریهای خودایمنی با منشای ناشناخته است که شیوع آن در زنان نسبت به مردان3 به1 است. این بیماری رابطه مستقیم با آترواسکلروزیس دارد و بیماریهای قلبی‌عروقی نصف علل مرگ‌ومیر در RA می‌باشد. براساس تحقیقات اخیر، احتمالا مکمل‌های آنتی اکسیدانی با کاهش استرس اکسیداتیو در کاهش چربی‌های سرمی و پیشگیری از آترواسکلروزیس نقش مهمی دارند. هدف از این مطالعه تعیین اثر مکمل ترکیبی آنتی اکسیدانی بر چربی‌های سرمی در RA می‌باشد.

Title: تعیین حساسیت، ویژگی و نقطه برش آزمون دیداری- حرکتی بندرگشتالت در تشخیص آسیب مغزی تروماتیک

http://yafte.lums.ac.ir/article-1-1116-fa.html

مقدمه: آزمون بندرگشتالت یکی از معروف‏ترین آزمون‏های نوروپسیکولوژیک است که ساده و سهل الاجرا بوده و جهت ارزیابی آسیب‏های مغزی مورد استفاده قرار می‏گیرد. هدف از این مطالعه تعیین میزان حساسیت، ویژگی و نقطه برش این آزمون در افراد دچار آسیب مغزی تروماتیک (TBI) بود. بحث و نتیجه‌گیری: نتایج این پژوهش نشان داد که آزمون بندرگشتالت در تشخیص آسیب مغزی تروماتیک خفیف نسبتاً ضعیف ظاهر می شود. ولی ویژگی آن بالا بوده و در تشخیص افراد سالم با موفقیت عمل می‏کند. یافته‌ها: در این پژوهش برای نقطه برش5/6، حساسیت 8/55%، ویژگی2/81 % و ناحیه زیر منحنی راک 69/0 محاسبه گردید. همچنین ارزش اخباری مثبت، ارزش اخباری منفی و کارایی به ترتیب برابر با 08/95%، 03/22% و17/59% به دست آمد. مواد و روش‌ها: در مجموع 120 بیمار دچار TBI با میانگین سنی 60/13± 25/31 در قالب یک مطالعه توصیفی- تحلیلی به شیوه نمونه‏گیری غیر احتمالی و پیاپی وارد مطالعه شدند. از همه بیماران ارزیابی‏های عصب‌شناختی توسط برش‌نگاری رایانه‏ای (CT اسکن)صورت گرفت و سپس آزمون دیداری- حرکتی بندرگشتالت به عمل آمد. برای تحلیل داده‏ها از آزمون منحنی راک استفاده گردید.

Title: ترکیبات KIR-HLA و استعداد ابتلا به سل

http://yafte.lums.ac.ir/article-1-1117-fa.html

مقدمه: سل (TB) که به وسیله مایکوباکتریوم توبرکلوزیس(Mtb) ایجاد می‌گردد، با حدود 9 میلیون مورد جدید و تقریباً 2 میلیون مرگ در هر سال، یک معضل مهم سلامتی عمومی در سراسر دنیا است. عموماً پاسخ‌های ایمنی انسان از گسترش Mtb جلوگیری می‌کند و عفونت در یک حالت نهفته باقی می‌ماند. هر دو پاسخ‌های ایمنی ذاتی و اکتسابی علیه TB درگیر می‌باشند. با این وجود نقش ایمنی ذاتی هنوز به خوبی مشخص نشده است. سلول‌های کشنده طبیعی (NK) از طریق مکانیسم‌هایی از قبیل سایتوتوکسیسیتی و تولید سایتوکاین، در میان اولین خط دفاع علیه عفونت‌ها می‌باشند. توانایی سایتوتوکسیسیتی NK به پذیرنده‌های شبه ایمونوگلبولینی سلول کشنده (KIR) موجود بر روی سطح سلول مربوط می‌شود. مجموعه ژنی KIR بر روی کروموزوم 19 در کمپلکس پذیرنده لکوسیت قرار دارد. پروتئین‌های KIR به‌عنوان پذیرنده‌هایی عمل می‌کنند که مولکول‌های آنتی‌ژن لکوسیتی انسان (HLA) کلاس I را شناسایی می‌کنند و به ‌طور مستقیم در فعالیت و مهار سلول‌های NK درگیر می‌باشند. KIRها و لیگاندهای HLA کلاس I آنها در پاتوژنز انواع مختلف بیماری‌ها مشارکت دارند. عدم تعادل KIRهای مهاری و فعال‌کنندگی، عامل کلیدی است که می‌تواند پاتوژنز TB را تحت تأثیر قرار ‌دهد. با این وجود نقش ترکیبات KIR-HLA در تعیین استعداد ابتلا به TB یک موضوع قابل بحث است. این مقاله مروری خصوصیات اصلی این ژن ها را خلاصه کرده و بحث می کند که چگونه ممکن است آنها در پاتوژنز TB درگیر باشند.
